# Protein Engineering Approaches to Enhance Fungal Laccase Production in *S. cerevisiae*

**DOI:** 10.3390/ijms22031157

**Published:** 2021-01-25

**Authors:** Pablo Aza, Felipe de Salas, Gonzalo Molpeceres, David Rodríguez-Escribano, Iñigo de la Fuente, Susana Camarero

**Affiliations:** Microbial & Plant Biotechnology Department, Centro de Investigaciones Biológicas Margarita Salas (CIB), CSIC, Ramiro de Maeztu 9, 28040 Madrid, Spain; pabloaza@cib.csic.es (P.A.); salasdelacuadra@gmail.com (F.d.S.); gonzalo.molpeceres@cib.csic.es (G.M.); david.rodriguez@cib.csic.es (D.R.-E.); idelafuente011@gmail.com (I.d.l.F.)

**Keywords:** laccase, heterologous production, *S. cerevisiae*, directed evolution, signal peptide, *N*-glycosylation, consensus design, synonymous mutations

## Abstract

Laccases secreted by saprotrophic basidiomycete fungi are versatile biocatalysts able to oxidize a wide range of aromatic compounds using oxygen as the sole requirement. *Saccharomyces cerevisiae* is a preferred host for engineering fungal laccases. To assist the difficult secretion of active enzymes by yeast, the native signal peptide is usually replaced by the preproleader of *S. cerevisiae* alfa mating factor (MFα1). However, in most cases, only basal enzyme levels are obtained. During directed evolution in *S. cerevisiae* of laccases fused to the α-factor preproleader, we demonstrated that mutations accumulated in the signal peptide notably raised enzyme secretion. Here we describe different protein engineering approaches carried out to enhance the laccase activity detected in the liquid extracts of *S. cerevisiae* cultures. We demonstrate the improved secretion of native and engineered laccases by using the fittest mutated α-factor preproleader obtained through successive laccase evolution campaigns in our lab. Special attention is also paid to the role of protein N-glycosylation in laccase production and properties, and to the introduction of conserved amino acids through consensus design enabling the expression of certain laccases otherwise not produced by the yeast. Finally, we revise the contribution of mutations accumulated in laccase coding sequence (CDS) during previous directed evolution campaigns that facilitate enzyme production.

## 1. Introduction

Laccases are multicopper oxidases that catalyse the oxidation of lignin phenols, aromatic amines and other various organic and inorganic compounds by reducing oxygen to water. Laccases are usually composed of three structural cupredoxin-like domains (D1–D3) each folded into a Greek key β-barrel topology [1]. Four copper ions (namely T1–T3) are located in two catalytic sites. The mononuclear T1 (blue copper) is located in D3 and serves as the primary electron acceptor site for substrate oxidation; the mononuclear T2 and binuclear T3 copper ions form the trinuclear cluster at the interface between D1 and D3 and function as electron acceptors from T1 prior to the reduction of oxygen [2,3,4].

Most basidiomycete laccases are monomeric glycoproteins carrying short mannose-enriched glycans linked mostly through *N*-glycosylation, which accounts for 5–25% of the Mw of the secreted enzyme. Although the number of putative *N*-glycosylation sites can vary among laccase sequences (according to the presence of the consensus Asn-X-Thr/Ser amino acid sequence), on average fungal laccases are *N*-glycosylated in 2–5 sites [5,6,7].

The high redox potential of laccases secreted during lignin degradation by wood-rot and litter-decomposing basidiomycete fungi makes these enzymes particularly suited to catalyse the oxidation of a wide range of substrates (phenols, amines, organic dyes, etc) of interest for different industrial sectors [8]. Nevertheless, their remarkable biotechnological potential is hampered by the difficult heterologous expression of basidiomycete laccases, making their study and industrial production challenging. Fungal laccases have been expressed in yeast such as *Saccharomyces cerevisiae* [9,10,11,12], *Kluyveromyces lactis* [13], *Yarrowia lipolytica* [14] and *Pichia pastoris* [15,16,17], and filamentous fungi like *Trichoderma reesei* [18], *Aspergillus oryzae* [19] and *Aspergillus niger* [12,20]. All of them comply with the essential protein post-translational modifications required for the functional expression of recombinant eukaryotic proteins (e.g., formation of disulfide bonds, glycosylation, etc). In addition to its capability to produce and secrete biologically active eukaryotic proteins, *S. cerevisiae* offers simple growth requirements, with low nutritional demands and short average production time, together with a well-annotated genome, genetic tractability and easy genetic manipulation. What is more, its high frequency of homologous DNA recombination makes this yeast an excellent expression system for enzyme-directed evolution [21]. However, most frequently the protein yields provided by *S. cerevisiae* are barely sufficient [22].

Recombinant protein production in *S. cerevisiae* can be promoted by taking advantage of the vast synthetic biology tools developed in this yeast and the availability of genetic elements (promoters, selectable markers, vectors) to design ad hoc expression systems. Other approaches to facilitate the secretion of foreign proteins to the extracellular environment include fine-tuning of post translational modifications like glycosylation or the utilization of yeast secretion factors [23].

Glycosylation plays an essential role in eukaryotic proteins as a major post-translational modification required for correct protein location, folding and biological activity [23]. The N-linked carbohydrate chains in laccases secreted by saprophytic basidiomycete fungi have been described to protect the enzymes from proteases produced by the fungus during wood degradation [24]. Preservation of *N*-glycosylation in several critical sites in fungal laccases has been found to support structural integrity by increasing persistent backbone hydrogen bonds to the protein surface [25]. On the other hand, glycosylation can be a bottleneck for heterologous protein production since differences in the glycosylation pattern between the wild and heterologous host may also result in miss activity of the recombinant protein. For instance, hyperglycosylation, especially excessive mannosylation [26], has been frequently described during production of heterologous proteins in *S. cerevisiae*, which could affect production titres.

Since the first work in 1983 [27], the preproleader sequence of the α-factor mating pheromone of *S. cerevisiae* (MFα1) has been widely used to assist the secretion of recombinant enzymes in *S. cerevisiae* [12,28,29]. Furthermore, modification of this secretion peptide by site-directed mutagenesis or directed evolution has resulted in enhanced secretion of the recombinant protein [12,30,31,32]. For instance, coevolution of the α-factor preproleader fused to fungal laccases during enzyme-directed evolution has proved to boost laccase secretion thanks to the mutations accumulated in the signal peptide [12,28,32,33].

Here we present some protein engineering approaches we carried out to facilitate the heterologous production of different basidiomycete laccases by *S. cerevisiae*. These approaches comprise (i) the use of the best evolved signal peptides obtained in previous laccase-directed evolution campaigns, (ii) the engineering of new *N*-glycosylation sites in the enzyme, (iii) the consensus enzyme design to introduce conserved amino acid residues that can enhance protein folding and stability; together with a revision of (iv) mutations of certain residues of the protein sequence that were introduced by random mutagenesis and selected during the enzyme-directed evolution in other studies.

## 2. Results and Discussion

### 2.1. Use of Evolved α-Factor Preproleader Sequences to Raise Laccase Secretion

**Figure 1:**
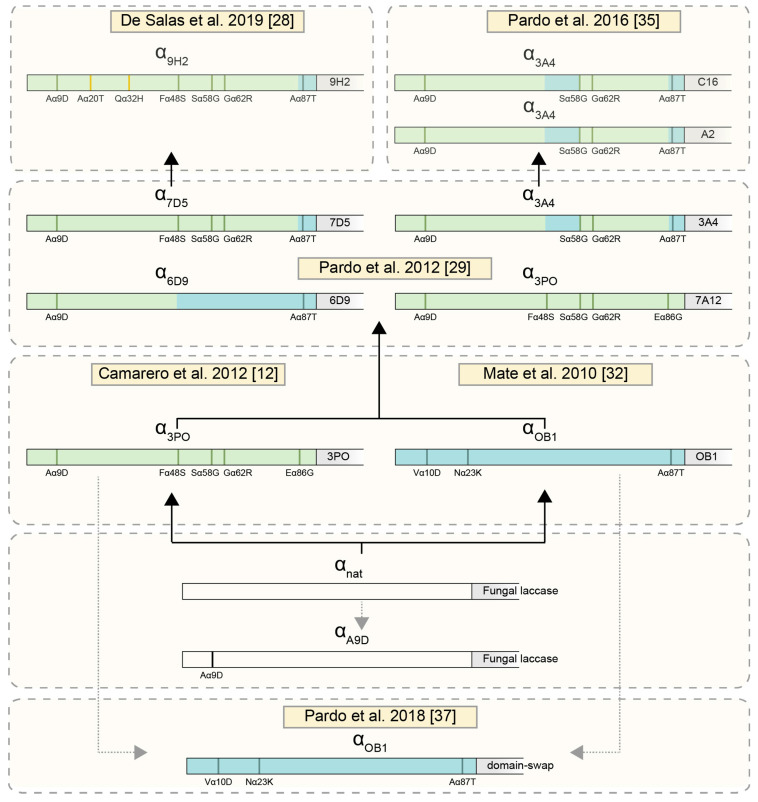
Scheme showing the mutations of the evolved α leaders tested in this study that were accumulated in the α_nat_, sequence (white) during successive laccase directed evolution campaigns in *S. cerevisiae*. The names of the evolved α leaders refer to the evolved laccase variant (in grey) with which they were first obtained. First, evolved laccases 3PO and OB1, and their leaders α_3PO_ (green) and α_OB1_ (blue) were respectively obtained during the directed evolution of *P. cinnabarinus* [12] and PM1 [32] laccases in *S. cerevisiae*. The mutations that arose in both evolution pathways are highlighted in green for α_3PO_ and blue for α_OB1_. Both evolved constructions were later recombined, giving rise to a pool of different chimeric signal peptides and laccases [29]. Afterwards, 3A4 laccase variant was used as template in Pardo and co-workers 2016, obtaining C16 and A2 laccases, both with α_3A4_ leader [35]. Parallelly, 7D5 was evolved to obtain the final fittest α_9H2_ leader, with the mutation Aα20T and Qα32H (indicated in yellow) [28]. The domain-swap laccase was designed by structure-guided DNA recombination to replace the second structural cupredoxin domain (D2) of OB1 laccase by that from 3PO, inheriting the α_OB1_ leader [37]. The α_A9D_ leader was obtained in the current work by site-directed mutagenesis over α_nat_ (white) as is described in the Materials and Methods section.

Directed evolution in *S. cerevisiae* of fungal laccases fused to the α-factor preproleader resulted in mutated laccase CDS and mutated α leader sequences that notably promoted enzyme secretion by the yeast [12,28,32,33]. For instance, during the directed evolution of *Pycnoporus cinnabarinus* laccase (PcL) for its functional expression in *S. cerevisiae*, several mutations accumulated in the evolved leader (α_3PO_) raised 40-fold the secretion of native PcL compared with the levels obtained with the native α leader (α_nat_) [12] from Invitrogen [34]. Then, the evolved α_3PO_ leader was later mutated in successive laccase-directed evolution campaigns [28,29,35], giving rise to α_9H2_ leader, which differs in seven mutations (Aα9D, Aα20T, Qα32H, Fα48S, Sα58G, Gα62R, Aα87T) from the α_nat_ leader (Figure 1). The α_9H2_ leader contributed to obtaining the highest yields reported so far for a basidiomycete laccase produced in *S. cerevisiae* [28]. We evaluate here the secretory potential of α_9H2_ leader as a signal peptide for production in *S. cerevisiae* of various laccases engineered in our lab. We used as references their own evolved signal peptides (Figure 1), since they have already been shown to improve laccase secretion as compared with the α_nat_ leader [12,36].

**Figure 2:**
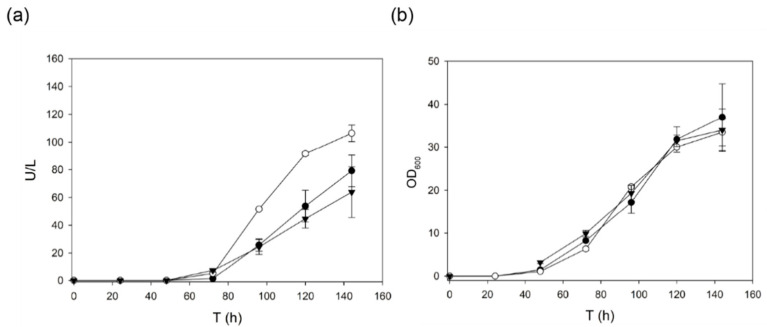
(**a**) Production of domain-swap laccase by *S. cerevisiae* flask cultures using its own evolved ⍺_OB1_ leader (black triangles), another evolved leader, ⍺_3PO_ (black circles), or the evolved ⍺_9H2_ leader lately obtained in our lab (white circles). (**b**) Yeast growth (OD 600).

First, we assayed the evolved α_9H2_ leader with the domain-swap laccase, a thermophilic enzyme remarkably stable at high temperature and in organic co-solvents. This enzyme had been designed by structure-guided DNA recombination to replace the second structural cupredoxin domain (D2) of OB1 laccase, obtained during the directed evolution of PM1L for functional expression in *S. cerevisiae* [32], by that of 3PO laccase (evolved from PcL) [37]. Domain-swap laccase inherited the leader from OB1 laccase (α_OB1_ leader). We compared the production of the enzyme with its own evolved α_OB1_ leader, with α_9H2_ leader, and with α_3Po_ leader as one of the first and more improved leaders [12,36]. Since domain-swap laccase is barely produced [12], we dropped the fermentation temperature to 20 °C to facilitate laccase secretion by slowing down cell growth. While the OD600 was identical in all *S. cerevisiae* flask cultures, laccase activities detected in the liquid extracts with α_9H2_ leader were 1.3-fold and 1.6-fold higher than those obtained with α_3PO_ and α_OB1_, respectively (Figure 2).

**Figure 3:**
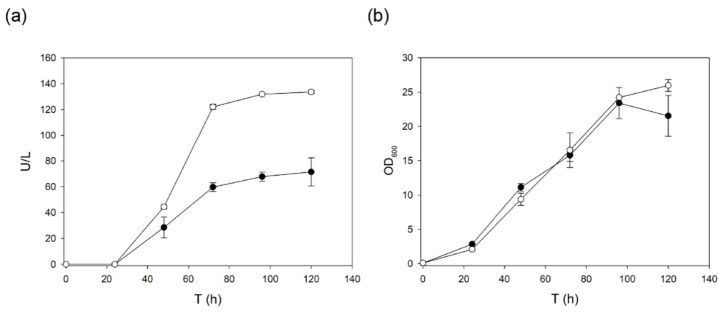
(**a**) Production of 3PO laccase by *S. cerevisiae* flask cultures (28 °C) using its own evolved ⍺_3PO_ (black circles) or the final fittest evolved ⍺_9H2_ leader (white circles). Laccase activity is depicted as U/L measured with ABTS, pH 3. (**b**) Yeast growth (OD 600).

We later confirmed the better performance of α_9H2_ leader over α_3PO_ leader for the secretion of 3PO laccase during *S. cerevisiae* flask fermentations, this time at 28 °C. Again, both fermentations showed quite similar OD600 curves, suggesting the irrelevant effect of the signal peptide on cell growth. By contrast, laccase production by *S. cerevisiae* was 2-fold enhanced when the enzyme was fused to α_9H2_ leader (Figure 3).

**Figure 4:**
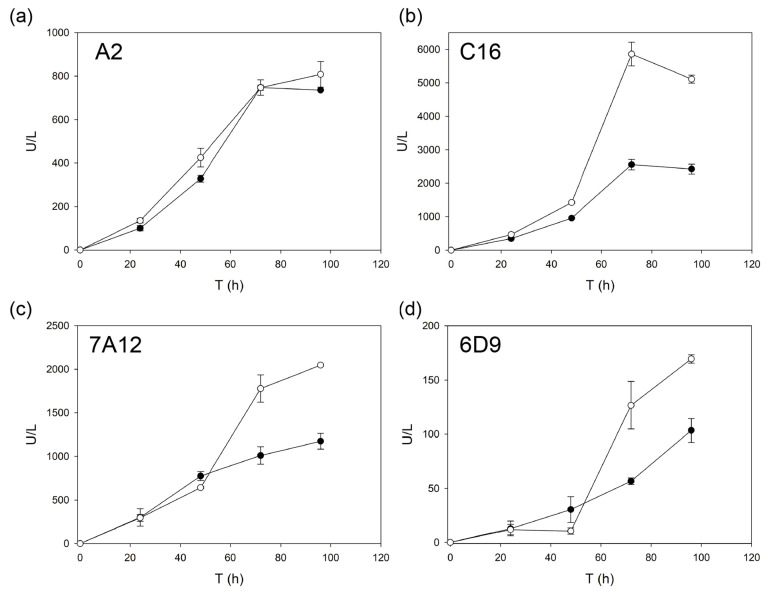
Production of the engineered laccase variants A2 (**a**), C16 (**b**), 7A12 (**c**) and 6D9 (**d**) using their own evolved ⍺ leaders (⍺_3A4_, ⍺_3PO_ or ⍺_6D9,_ black circles) as depicted in Figure 1, or the final evolved ⍺_9H2_ leader (white circles) by *S. cerevisiae* flask cultures.

The superior secretory potential of α_9H2_ leader for laccase production in *S. cerevisiae* was thereafter compared with other α leader sequences obtained in our lab during other laccase evolution campaigns. For that, we assayed the production of the following laccase engineered variants: 7A12 and 6D9 [29], A2 and C16 [35], fused either to α_9H2_ or to their corresponding evolved ⍺ leaders (see Figure 1). In general, laccase activities obtained with α_9H2_ leader were significantly higher, except for A2 laccase, where both signal peptides gave similar results (Figure 4).

**Figure 5:**
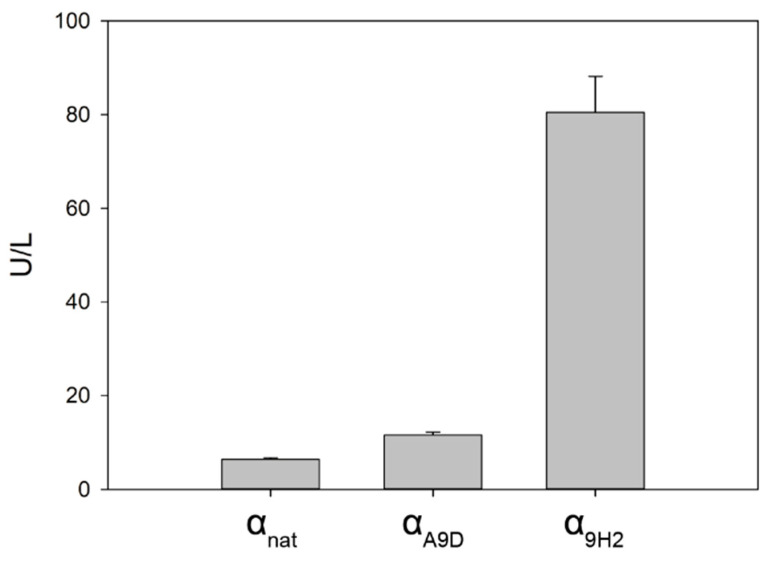
Production of *Argocybe pediades* lacasse (ApL) fused to either the fittest evolved ⍺_9H2_ leader, the native ⍺_nat_ leader or the mutated ⍺_A9D_ leader by *S. cerevisiae* after 48 h of fermentation in microplates in SEM.

Since all the aforementioned laccase variants originated from the same parent laccases (PcL and PM1L, both from Polyporales strains), we aimed to evaluate the secretory potential of ⍺_9H2_ leader to aid the production of laccases from other basidiomycete sources. Two laccases from Agaricales fungi, *Agrocybe pediades*, ApL (ID 823,363 JGI), and *Pleurotus eryngii*, PeL (ID 152,153 JGI), were assayed. PeL belongs to the recently classified NLAC that constitute a separate cluster of laccase-like enzymes that are not found in Polyporales [38], whereas ApL is a sensu stricto laccase. The CDS of both laccases were synthesized de novo for expression in *S. cerevisiae*, replacing the predicted native signal peptides by the ⍺-factor preproleader, in particular by ⍺_nat_ and ⍺_9H2_ leaders. Additionally, we included in the comparison the mutated ⍺_A9D_ leader, which holds mutation A9D in the preregion. This mutation (present in ⍺_3PO_ leader) had been demonstrated to be responsible for a remarkable improvement of laccase secretion by *S. cerevisiae* during the directed evolution of PcL [12]. Additionally, a similar mutation in the hydrophobic core of the canonical preregion (V10D) was selected during PM1L engineering [32], and several substitutions on this core also enhanced the production of immunoglobulin [30], evidencing its influence in the secretion potential of the signal peptide. The performance of the two mutated signal peptides, ⍺_9H2_ and ⍺_A9D,_ were compared with that of ⍺_nat_ leader in *S. cerevisiae* microcultures, and, in order to facilitate laccase detection, we used a minimal expression medium (SEM) to avoid the background of the rich expression medium (EB) used in flask cultures [36]. Detectable ApL activities were obtained with the three ⍺ leaders in the supernatants of *S. cerevisiae* microcultures as follows: ⍺_nat_ < ⍺_A9D_ << ⍺_9H2_ (Figure 5). By contrast, no activity could be detected for PeL with any of the three ⍺ leaders.

All these results confirmed the suitability of utilising evolved α leaders, in particular α_9H2_, as signal peptides to promote the heterologous production of fungal laccases by *S. cerevisiae*.

### 2.2. Engineering of New N-Glycosylation Sites in the Enzyme

**Figure 6:**
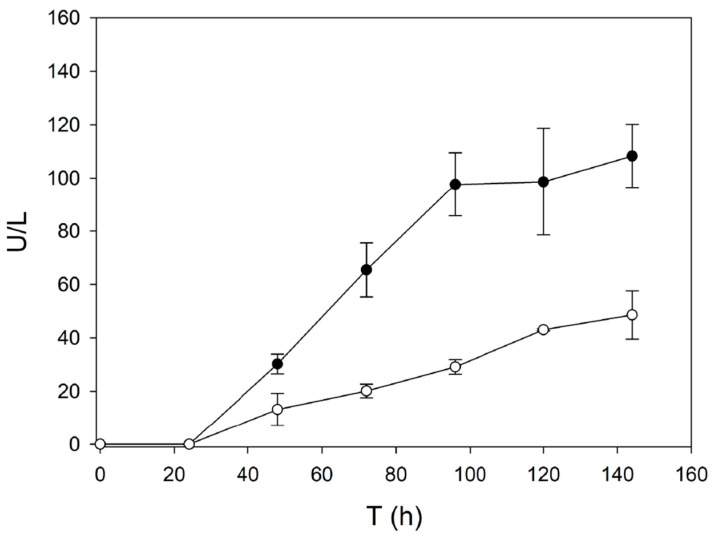
Production of domain-swap laccase (black circles) and its deglycosylated variant N215G (white circles) by *S. cerevisiae* cultured in flask. In both cases the evolved ⍺_9H2_ leader was used as signal peptide.

The domain-swap laccase has three *N*-glycosylation sites: N54 and N433 located in the first (D1) and third (D3) cupredoxin domains of OB1 laccase evolved from PM1L (see PDB ID: 5ANH [19]), and N215 inherited from D2 of 3PO laccase evolved from PcL (see PDB ID: 2XYB). The latter *N*-glycosylation site seems to be responsible for the hyperglycosylation of the enzyme by *S. cerevisiae*. The contribution of N215 site as possible determinant for the outstanding thermostability of swap-domain laccase was evaluated in the deglycosylated variant N215G. It was proved that extra glycosylation in this site was not responsible for the improved thermostability of the enzyme [37]. On the contrary, removal of the N215 site strictly diminished the laccase activity detected in the supernatants of *S. cerevisiae* cultured in flasks (Figure 6), suggesting a possible role of *N*-glycosylation in the heterologous production of the enzyme.

**Figure 7:**
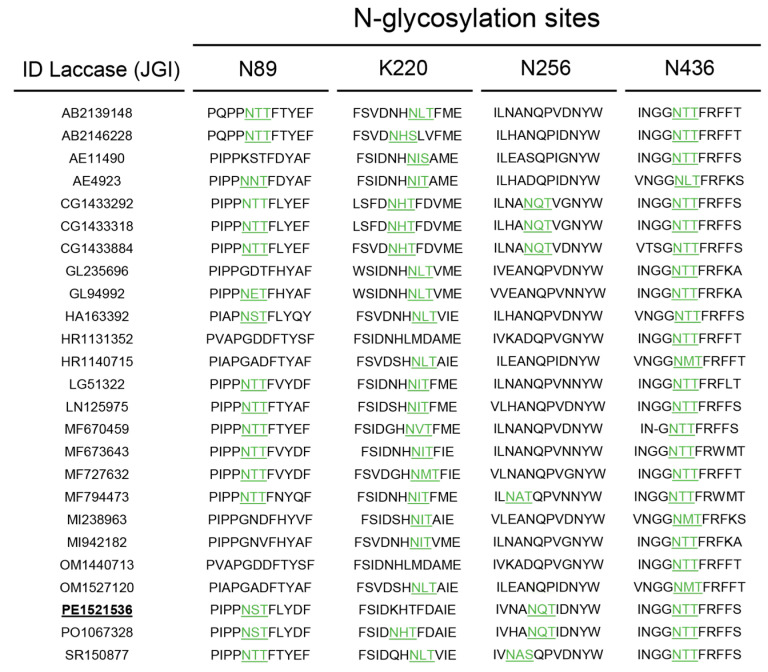
Sequence alignment of the 25 NLAC found in Agaricomycotina compared with *Pleurotus eryngii* laccase (PeL) sequence (PE1521536 in bold, underlined), showing the NXT/S motifs that account for putatively conserved *N*-glycosylation sites (in green, underlined).

**Figure 8:**
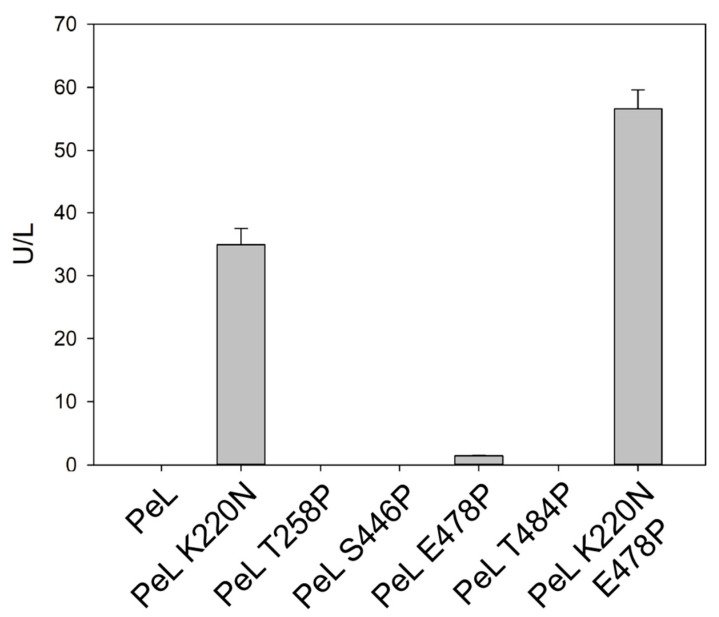
Production of PeL and its glycosylation variant (PeL K220N) and other PeL variants built by replacing certain residues by consensus Pro. The evolved ⍺_9H2_ leader was used as signal peptide in all cases. Laccase activities were measured with ABTS pH 3 after 48 h of *S. cerevisiae* fermentation in microplates in SEM.

Taking the aforesaid into account, we addressed the lack of expression of PeL by analysing the *N*-glycosylation sites in this enzyme. Three putative *N*-glycosylation sites were found in PeL: N89, N256 and N436, respectively located in domains D1, D2 and D3. We analysed the *N*-glycosylation sites in the 25 NLAC sequences found in the 52 fungal genomes previously studied [38]. We observed that N89 and N436 were largely conserved in NLAC sequences, as well as in basidiomycete laccases sensu stricto, whereas N256 was much less frequent. Conversely, the majority of NLAC held the N220 site, also located in D2 (Figure 7). Hence, we introduced by site-directed mutagenesis a new *N*-glycosylation site in 220 position of PeL in an attempt to improve the particularly difficult expression of the enzyme in *S. cerevisiae*. The ⍺_9H2_-PeL construction was used as a template to introduce the PeL K220N variant and their heterologous expression was tested in *S. cerevisiae* microcultures. The additional *N*-Gly site introduced in PeL enabled the functional expression of the enzyme by *S. cerevisiae*, detecting significant laccase activity levels in the supernatants of *S. cerevisiae* microcultures (Figure 8).

The N220 site introduced in PeL is analogous to the N215 site of the domain-swap laccase, and it is conserved in 40% of the Polyporales laccases studied [38], supporting the possible contribution of glycosylation in this site for proper laccase production. This N site is not present in *Agrocybe pediades* laccase (ApL). Nevertheless, from the three *N*-glycosylation sites predicted in ApL (N21, N255 and N439, each respectively located in a different laccase domain), N255 in D2 coincides with the aforementioned N256 site of PeL. When we removed N255 site from ApL variant by site-directed mutagenesis, the laccase activity found in *S. cerevisiae* flask fermentations was significantly reduced (over 6 times); in both cases, ⍺_9H2_ leader was used as signal peptide (Table S1).

The crucial role of certain *N*-glycosylation sites in laccases seems to be rather related to their location in the protein, which determines that its addition or removal cause a profound conformational change [7]. For instance, from the three *N*-glycosylation sites (N75D, N238D or N458D) of *Lentinus* Lcc4 (produced in *Pichia pastoris)*, the removal of glycosylation in N238 and N458 sites caused a significant loss of activity detected in the yeast culture supernatants. The N458 site is highly conserved (N439 in ApL and N436 in PeL), whereas the N238 site is analogous to the conserved N220 in NLAC. In *Lentinus*, laccase glycans linked at N458 (located in D3) and N238 sites (in D2) interact directly with a lengthy loop which crosses over the two laccase domains, connecting D2 and D3. The authors hypothesised that the H-bond networks between the loop and the glycan moieties play a crucial role on protein activity that was severely reduced with the removal of these *N*-glycosylation sites [7]. However, in our opinion it is difficult to discriminate whether this is the result of inferior catalytic activity or reduced enzyme production, since no kinetic data were provided. Nevertheless, the results obtained here by introducing the new N220 site in PeL and removing N215 in domain-swap laccase (both analogous to N238 in *Lentinus* Lcc4) coincide to point out the crucial role of *N*-glycosylation in this site. Moreover, it seems that laccases lacking the N238 site present nearby alternative glycosylation sites in D2 such as N255 in ApL, whose removal drastically reduced the laccase activity detected in *S. cerevisiae* cultures (Table S1). To assess the real contribution of glycan anchoring in this position to enzyme activity or production, we purified ApL variants before and after removal of the N255 site for their characterisation. SDS-PAGE of both ApL variants demonstrated that the N255 site is not putative but a real *N*-glycosylation site and, therefore, its removal produced a deglycosylated variant (Figure S1). Additionally, when we measured their catalytic constants, we observed a detrimental effect on enzyme activity in the deglycosylated variant (*k_cat_* was reduced 2-fold) (Table S2). However, this only partially explained the 6-fold diminished activity detected in the liquid extracts as compared with the non-deglycosylated variant (Table S1). Thus, the positive effect of glycosylation in D2 for laccase production by *S. cerevisiae* was evidenced, as well as in laccase catalytic activity.

Actually, the influence of *N*-glycosylation on the functionality of fungal laccases is a complex issue not yet fully understood. It seems to have a substantial role in fine-tuning enzymatic properties such as catalytic activity in wild laccases produced by basidiomycete strains [39,40]. On the other hand, changes in the glycosylation pattern through laccase heterologous expression in yeast have been reported to either improve enzyme stability, or change substrate affinity or activity [41,42] due to the addition of large glycan chains to the protein backbone. However, in our experience, laccase hyperglycosylation by *S. cerevisiae* does not contribute to improve the stability of the enzyme [37], in agreement with other studies [7]. Moreover, hyperglycosylation of fungal laccases by this yeast occurs only with certain laccases and is not necessarily correlated with a decrease of secreted enzyme activity coinciding with other results [43]. In fact, here we provide evidence that addition of new *N*-glycosylation sites can stimulate the production of properly folded and active laccases by *S. cerevisiae*, in concordance with already reported production of other heterologous proteins [44], especially when they are impaired in secretion due to aggregation [45].

### 2.3. Consensus Enzyme Design

The introduction of the N220 site in PeL is part of the consensus design we followed in an attempt to facilitate the difficult heterologous expression of the enzyme by exploiting the evolutionary information encapsulated in homologous NLAC sequences [38]. Consensus protein design is based on the hypothesis that, at a given position, the respective consensus amino acid contributes more than the average to the stability of the protein than non-conserved ones [46]. It has shown high success rates in creating well-folded and stable proteins that retain biological activities.

**Figure 9:**
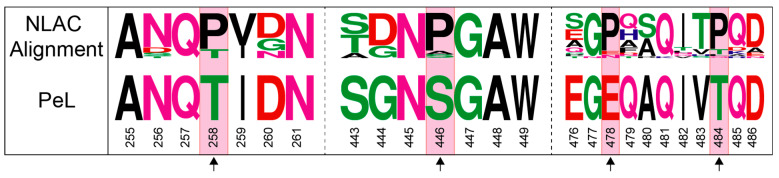
Sequence logo for the 25 NLAC sequences found in Agaricomycotina compared with PeL (NLAC) sequence showing the conserved surface Pro that were, respectively, introduced in PeL through mutations T258P, S446P, E478P, T484P.

**Figure 10:**
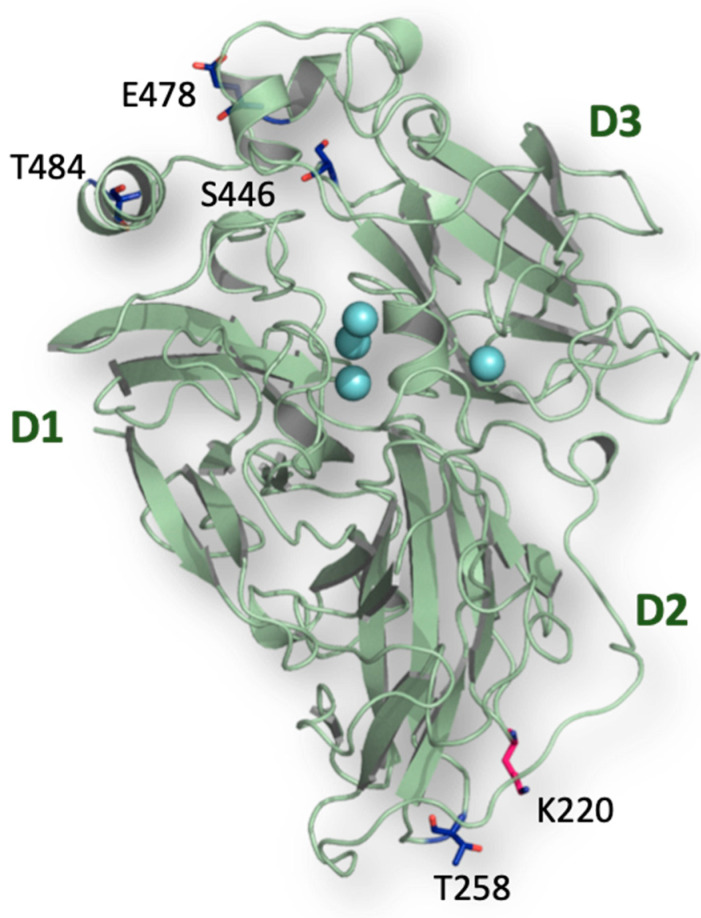
Structure of PeL (modelled with PDB ID: 3PXL as the template) showing the location of the residues that were mutated to introduce a consensus *N*-glycosylation site (K220N) and several consensus Pro residues on the surface of the protein through mutations T258P, S446P, E478P and T484P.

The mature sequence of PeL was compared with the consensus sequence obtained from the multiple alignment of the 25 NLAC sequences found in 52 Agaricomycotina genomes [38]. We searched for putatively conserved *N*-glycosylation sites and proline residues that were absent in PeL. We detected one consensus Asn residue participating in a putatively conserved *N*-glycosylation site (Figure 7) and selected four consensus proline residues (Figure 9) placed in the protein surface in positions distant from the active site (Figure 10). These amino acid residues were individually introduced in PeL through site-directed mutagenesis to obtain the single mutated PeL K220N, T258P, S446P, E478P and T484P variants that were fused to ⍺_9H2_ and expressed in *S. cerevisiae*. In addition to the aforesaid remarkable effect of mutation K220N (see previous subsection), consensus mutation E478P also enabled to obtain detectable laccase activity levels in the supernatants of the yeast microcultures. Conversely, none of the other single mutations provided detectable laccase activities (Figure 8). We combined mutations K220N and E478P in PeL and observed a positive synergism between both mutations that was reflected in the higher levels of activity secreted by *S. cerevisiae* microcultures (Figure 8) and in the superior thermostability of the double-mutated variant in comparison with PeL K220N (Table 1).

**Figure 11:**
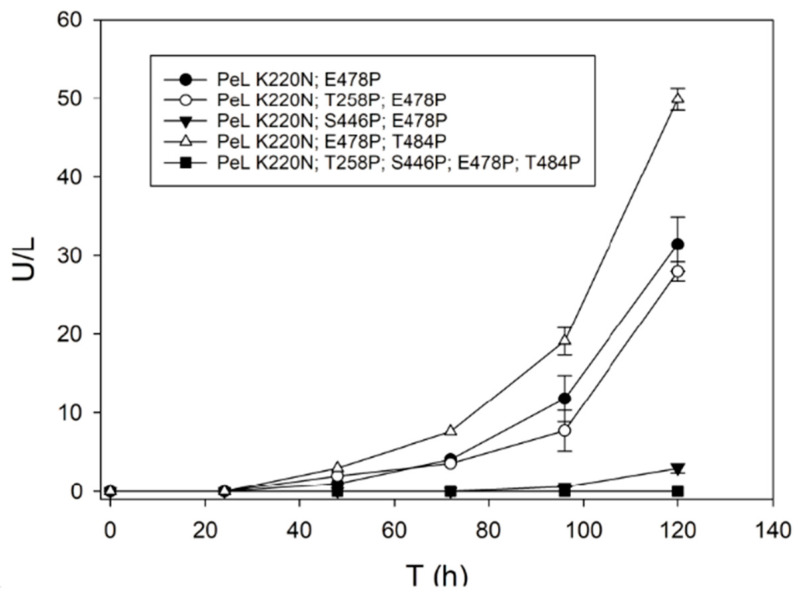
Production by *S. cerevisiae* cultured in flask of the double-, the three triple- and the quintuple-PeL mutated variants obtained by consensus design. All laccase sequences were fused to ⍺_9H2_ leader as signal peptide.

Then, we constructed over PeL K220N, E478P (i) three triple mutants by introducing separately mutations T258P, S446P or T484P, and (ii) a quintuple mutant by introducing the three mutations together. The production curves of the multiple variants by *S. cerevisiae* flask cultures were compared with the production of the double-mutated variant (Figure 11). The quintuple-mutated variant rendered almost undetectable laccase activity. Introduction of mutation T258P in the corresponding triple variant did not significantly affected enzyme activity levels as compared with the double-mutated variant, whereas S446P severely impaired them. The latter mutation also strongly reduced laccase thermostability (Table 1). By contrast, we found a notable increment of laccase activity levels for the PeL K220N, E478P, T484P variant, although the thermostability of the enzyme was somehow reduced. The double- and triple-mutated laccase variants were stable at neutral and alkaline pH but much less stable at pH 3 (Table 1).

The molecular mechanisms involved in protein stability include, among others: disulphide bridges, ion pairs, hydrogen bonds, hydrophobic interactions, packing, decrease of the entropy of unfolding state and inter-subunit interactions [47]. After consensus design, enzymes show improved thermodynamic stability and increased robustness of the native structure to assure the minimal stability required to fold [48,49]. We were not able to determine if consensus N220 site improved the thermal stability of PeL due to the lack of expression of the native enzyme. However, glycosylation in the N220 site seems to have a positive impact on the production of the folded enzyme, in agreement with that found for *Lentinus* lcc4 [7] (see previous subsection). On the other hand, Pro is the amino acid with the lowest conformational entropy due to the rigidity of the pyrrolidone ring. The introduction of consensus proline residues would decrease the backbone entropy of the PeL unfolding state, thus contributing to the increase in the free energy change for protein thermostabilization [50]. However, not every Pro introduced in PeL stabilised the enzyme, most probably due to the different environments of the mutated sites. This fact agrees with recent consensus design of OB1 laccase where several consensus mutations incremented thermostability and secretion, but others resulted neutral and deleterious [51].

**Figure 12:**
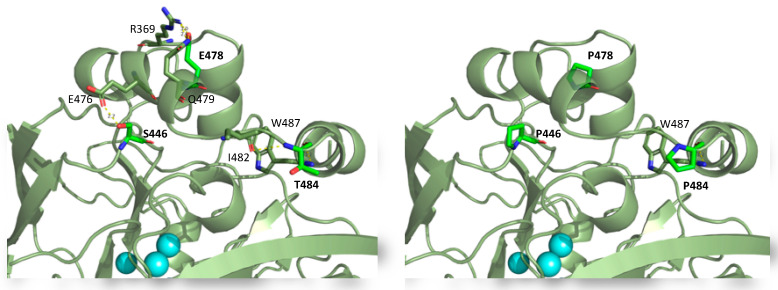
Detail of PeL structure model showing the polar contacts of residues 446, 478 and 484 before and after being mutated to consensus Pro.

It has been estimated that the stability of the native form of a protein increases by about 2–4 kJ/mol when a proline residue is introduced into a protein chain at a location that does not alter the protein structure [50]. However, if proline accommodation imposes some regional strain or unfavourable steric contact, it results in protein destabilization [52,53]. The three mutations S446P, E478P and T484P introduced in PeL reduced the number of polar interactions with neighbour residues (Figure 12), although they produced dissimilar effects. Prolines 446 and 484 were introduced in a loop and in the N-terminus of an α helix, respectively; both are common locations for proline in proteins. However, Pro 446 had a strong negative impact on the thermostability and production/activity of the enzyme, whereas Pro 484 notably boosted the laccase activity levels secreted in *S. cerevisiae* cultures (Figure 11 and Table 1). Conversely, laccase production and thermostability were significantly raised by the consensus Pro 478, despite it interrupting a putative salt bridge between E478 and R369 and one H bond with Q479. In addition, the location of Pro 478 in the middle of an α helix is considered as a destabilizing feature of protein structure. The change in free energy of folding for introducing Pro in α helix is about 14 kJ/mol [54], and the presence of proline residues in an α-helix has been regarded as problematic because of their ability to break its structure (there are several reports of proline mutations as a pathogenic mechanism) [55].

Nevertheless, even when the kinked proline-containing helix can be considered a rare feature, they are not all that uncommon in some globular proteins (e.g., transmembrane proteins), where a range of kink angles and a variety of hydrogen bonding schemes have been found [54], suggesting a function related to conformational flexibility [56]. Prolines in α-helices are one of the unique characteristics of karyopherins and some other HEAT repeat-containing proteins able to pass through the amphiphilic matrix of the nuclear pore complex [57]. In karyopherins, proline does not serve as a “breaker” of α-helical proteins, but rather behaves as a “protector” of the flexible molecular conformation of the protein to achieve efficient nuclear transport [58]. The local destabilization of α-helices finally contributes to maintain the overall molecular structure. In line with this, we recently demonstrated that mutations of the laccase C-terminal producing a loosening of α helix secondary structure and increasing the mobility of the region, strongly improved the stability of the enzyme against thermal denaturation [28]. The higher flexibility of the C-terminal helped neutralize the destabilization caused by thermal fluctuations at high temperatures, which could allow the rest of the protein to maintain the native structure and remain active.

Finally, since proline can exist in cis- and trans-configuration, the contribution of a specific proline residue to protein stability is associated with the thermodynamic equilibrium between cis- and trans-isomers of the peptide bond between Pro and its preceding residue [59]. In PeL in particular, a Gly residue (strictly conserved in NLAC) precedes Pro 478 (Figure 9). Glycine appears with high propensity at pre-cisPro positions in proteins, rescuing secondary structures from severe distortions. The Gly-cisPro motif is evolutionarily conserved, functionally important and dynamic in nature [60,61].

### 2.4. Mutations Accumulated in the Protein CDS during Directed Evolution

Mutations accumulated in the protein CDS during directed evolution in *S. cerevisiae* might be advantageous for the heterologous expression of the recombinant enzyme. Some of these mutations are synonymous mutations that remain at the nucleotide level, without affecting the amino acid sequence of the protein, but can significantly influence protein abundance through changes in translation efficiency. On the one hand, they can provide a change to a synonymous codon more frequently used in the heterologous host. Synonymous mutations favouring codon usage in *S. cerevisiae* were selected during the directed evolution of fungal laccases from *Myceliophthora thermophila*, *Trametes sp*, PM1 basidiomycete and *P. cinnabarinus* [12,32,33,62,63] carried out in this yeast. The accumulation of synonymous mutations in the CDS of the final evolved enzymes would favour the bias toward a specific subset of codons (related to the levels of the corresponding tRNAs in the eukaryotic apparatus) and, consequently, speed up the elongation rate by avoiding translation pauses [64]. Another suggested mechanism by which synonymous mutations can modulate protein abundance is the folding energy of the mRNA transcript, which may influence ribosome binding and therefore translation initiation [65].

Substitutions of amino acid residues in the mature protein can also influence enzyme production by improving protein folding and maturation in addition to a possible contribution to protein robustness. Several beneficial mutations of amino acid residues exposed in the surface of distal protein regions, far away from the catalytic site, have been discovered during laccase engineering and associated with improvements in enzyme secretion by *S. cerevisiae*. For instance, during the in vitro evolution campaigns of laccases from PM1 basidiomycete [32] and *P. cinnabarinus* [12] toward functional expression by the yeast, we selected mutations in similar distal locations of D2, respectively, in residues Asp281 and Arg280. Both mutations induced conservative amino acid replacements (R280H and D281E), and both interrupted several hydrogen bonds with neighbour residues, thereby enhancing the flexibility of this region, which might facilitate protein folding during the post-translational stages.

During different enzyme evolution campaigns of *M. thermophila* laccase (MtL) in *S. cerevisiae*, mutation of residue 552 to Asn was repeatedly selected. This residue is located in the surface of the laccase, in a loop far away from the catalytic pocket. Asn was first selected during the directed evolution of the enzyme for expression in *S. cerevisiae*, through mutation Y552N [33]. Later on, during MtL evolution for the synthesis of polymeric dyes, mutation H552N was unexpectedly selected [63]. This mutation recovered the Asn that had been lost in an intermediate evolution campaign and contributed to obtaining a final production yield of 37 mg/L of this ascomycete laccase. Thus, the presence of asparagine in this position has been associated with the improved functional expression of the enzyme by this yeast (even it is not related to the addition a *N*-glycosylation site). Another recent example of a mutation placed in a flexible loop, far from the T1 site, and exposed to the protein surface, is that we recently selected in residue 159 during the directed evolution of *A. pediades* laccase. The mutation was responsible for a 3-fold increment in the activity detected in the liquid extracts of *S. cerevisiae* cultures. Since this mutation did not change the activity of the enzyme towards different substrates, it has been related with an improvement in enzyme production (unpublished data).

Finally, it is worth mentioning that mutations contributing to enzyme production are not always located in distal loops of the protein. This was the case of Phe 454 substitution to Pro located in the active site of RY2 laccase, which was developed in our lab through directed evolution and computational design and constitutes a robust biocatalyst for green chemistry [28]. Despite the F454P mutation being contiguous to His 455 ligand of T1 copper, it did not modify the kinetic activity of the enzyme. By contrast, it was associated with an increase of laccase production from 16 mg/L to 25 mg/L. This mutation was selected from the saturation mutagenesis of position 454. Even though different amino acid substitutions led to significant improvements in the detected activity, only Pro did not severely impair the stability of the enzyme, even when it was placed in the middle of an α helix [28].

## 3. Materials and Methods

### 3.1. Reagents and Strains

Yeast Transformation Kit was purchased from Sigma-Aldrich (St. Louis, MO, USA). High Pure Plasmid Isolation Kit and ABTS (2,2′azinobis (3ethylbenzothiazoline- 6 sulphonic acid)) were obtained from ROCHE. Phusion High-Fidelity DNA polymerase and Restriction enzymes (*Not*I and *Bam*HI) were obtained from New England Biolabs. QIAquick gel extraction kit from Qiagen (Hilden, Germany)). Zymoprep™ Yeast Plasmid Miniprep II was purchased from Zymo Research (Tustin, CA, USA). *S. cerevisiae* BJ5465 strain was purchased from LGC Promochem (Barcelona, Spain). The α-factor preproleader (α_nat_) was obtained from the pPICZα family of plasmids of Invitrogen (Waltham, MA, USA).

### 3.2. Culture and Media

Minimal Medium (MM) and EB expression medium were prepared as previously described [12]. SEM expression medium was prepared as previously described [36], without adding ethanol. Four or 2 mM CuSO_4_ were added for laccase expression in EB and SEM media, respectively.

### 3.3. Predictions and Modelling

Prediction of *N*-glycosylation sites was performed using the NetNGlyc 1.0 Server (http://www.cbs.dtu.dk/services/NetNGlyc/). The PeL 3D model was built with the Swiss-model server [66] using *Trametes hirsuta* laccase structure (PDB ID: 3PXL) as template. The analysis of mutations of the mature protein and representation of 3D protein structures were performed using PyMol. Putative signal peptide was analysed with SignalP-5.0 Server [67]. The graphical representations of amino acid frequency for the searching of consensus prolines were done with WebLogo [68].

### 3.4. Laccase Variants and Libraries Constructions in S. cerevisiae

#### 3.4.1. Signal Peptides

The CDS of *Pleurotus eryngii* laccase (JGI ID: 152153) and *Agrocybe pediades* laccase (ID 823,363 JGI) were optimized for codon usage in *S. cerevisiae* with the OPTIMIZER Web server using the random guided method [69] and synthesised de novo by ATG Biosynthetic fused to the sequence of α_nat_ leader. Laccases 7A12 [29], 6D9, A2 [35] domain-swap [37] and 3PO [12] were obtained from our collection of enzymes engineered in *S. cerevisiae*; all cloned in the uracil-independent and ampicillin-resistant vector pJRoC30 with their respective evolved α leaders.

Overlapping ends primers were designed for ApL and PeL cloning in pJRoC30 vector (Table S3). Two independent PCR reactions were carried out with the general ExtFw sense primer, and a specific antisense primer for PeL (PeL-pJRoC Rv) and ApL (ApL-pJRoC Rv). The pJRoC30 vector was digested with NotI and BamHI, and the resultant linearized vector was co-cloned with given PCR products to obtain the α_nat_-PeL and α_nat_-ApL constructions by In Vivo Overlap Extension (IVOE) in *S. cerevisiae* [70].

For fusing different signal peptides to different laccase variants, a first fragment was obtained by PCR with ExtFw sense primer and 87Final-Rv antisense for α_9H2_ or 86Final-Rv antisense for α_3PO_ leaders, and a second fragment was obtained by PCR with 87Final-Fw sense for α_9H2_ or 86Final-Fw sense for α_3PO_ and ExtRv antisense primer (Table S3). The two PCR fragments were co-cloned as explained above.

The α_A9D_-PeL and α_A9D_-ApL variants were obtained as follows: using their respective α_nat_ leader constructions two PCR were performed as aforesaid; a first fragment with ExtFw sense and A9D Rv antisense primers and a second fragment with A9D Fw sense and Ext Rv antisense primer. The two reaction products were co-cloned as explained.

#### 3.4.2. *N*-Glycosylation and Consensus Prolines

Mutagenic primers were designed so that single point mutations were introduced in PeL sequence by site-directed mutagenesis, using α_9H2_-PeL as template for the individual substitutions, α_9H2_-PeL K220N for obtaining the double variant (α_9H2_-PeL K220N; E478P), and the latter for obtaining the triple variants. For each mutagenesis site two fragments were obtaining; a first fragment with the ExtFw sense primer and the corresponding antisense mutagenic primer, and the second fragment with their specific mutagenic sense primer and the general ExtRv antisense primer (Table S3). Same process was followed for obtaining the deglycosylated variant N255 ApL.

### 3.5. Laccase Production

#### 3.5.1. Flask Fermentation

Three single *S. cerevisiae* colonies transformed with the plasmid containing a given laccase mutant were picked and inoculated in 3 mL MM and incubated for 48 h at 30 °C, 200 rpm. An aliquot of the culture was used to inoculate a final volume of 10 mL (MM) in 100 mL flask with a final OD600 of 0.3. After 4 h incubation at 30 °C (OD600 close to 1), cells were diluted to OD600 = 0.1 in 30 mL EB medium in 100 mL flask. Laccase activities secreted in the liquid extracts were measured by the oxidation of ABTS (ε418 = 36,000 M^−1^ cm^−1^) in citrate phosphate (CP) 100mM pH 3, using the UV-1900 Shidmazu spectrophotometer. Maximum activity was roughly reached after 4 days of incubation at 28 °C and 6 days at 20 °C. At that moment, cells were centrifuged at 13,000 rpm, 4 °C and the supernatants concentrated using 10 KDa AmiconUltra Centrifugal filters at 5000 rpm for 10 min.

#### 3.5.2. Microwell Production

Five colonies of each *S. cerevisiae* clone (transformed with a particular laccase variant) were picked in 96-well plates, containing 50 μL of MM per well, and grown at 28 °C, 225 rpm, and 80% relative humidity in a humidity shaker (Minitron-INFORS, Bottmingen, Switzerland). After 24 h, 160 μL of SEM medium was added to each well, and the plates were incubated at 28 °C for 48 h. The plates were centrifuged (Eppendorf 5810R centrifuge, Hamburg, Germany) for 5 min at 3000 *g* at 4 °C, and 20 μL of supernatant was mixed with 180 μL of 3 mM ABTS in 100 mM CP pH 3. The plates were briefly stirred and laccase activity was determined in kinetic mode by the increment in Abs 418 in a plate reader SpectraMax M2 (Molecular Devices, Sunnyvale, CA, USA).

### 3.6. Laccase Characterization

#### 3.6.1. pH Activity Profile

Optimum pH of crude enzymes was measured using 20 µL of the concentrated supernatants (at 0.1 U/mL) and 180 μL of 3 mM ABTS in 100 mM Britton Robinson (BR) buffer at pH 2–8 range. The solution was mixed and measured in kinetic mode in triplicate. Relative activities were calculated respecting the maximum activity of each laccase variant.

#### 3.6.2. Thermostability Assay

T50 is defined as the temperature at which the enzyme retains 50% of its activity after a period of incubation. Samples with 0.1 U/mL of crude or purified enzyme were incubated in a 30–80 °C temperature gradient in a thermocycler (in triplicate) for 10 min. After cooling the enzymes at 4 °C for 10 min and tempering at room temperature for another 10 min, 20 μL aliquots were mixed with 180 μL 3mM ABTS in 50 mM CP buffer pH 3 to determine laccase activity at 418 nm in kinetic mode. The thermostability values were calculated from the ratio between the residual activities incubated at different temperatures and the maximum activity.

#### 3.6.3. pH Stability Assay

Aliquots of 0.1 U/mL of crude or purified enzyme were incubated in 100 mM BR Buffer at different pH values (3–9) for 2, 4, 6 and 24 h. Residual activities at different times were measured with 20 μL samples and 180 μL 3 mM ABTS in 50 mM CP buffer pH 3, in triplicate, in kinetic mode. The relative activity was calculated as a percentage of the initial activity.

## 4. Conclusions

The difficult heterologous expression of fungal laccases is often a bottleneck for the study and application of these green biocatalysts. Enzyme-directed evolution allows to tailor enzymes with enhanced robustness or catalytic activities and increase production yields. Here, we demonstrate the outstanding ability of α_9H2_, a mutated α-factor preproleader obtained through successive laccase evolution campaigns in *S. cerevisiae*, to enhance laccase secretion by the yeast. Mutations of the enzyme sequence can also have a positive effect on enzyme production. We evidence here the role of *N*-glycosylation on laccase production and properties, and introduce consensus mutations in the protein scaffold that allow the functional expression of particularly recalcitrant enzymes. Finally, we revise the role of synonymous mutations that ease translation efficiency or of mutations of the protein scaffold favoring protein folding and maturation.

## Figures and Tables

**Table 1:** Thermostabilities as T50 (10 min) values and stabilities after 24 h incubation at different pH (indicated as % of the initial activities) of the PeL variants obtained by consensus design.

PeL Variant	T50 (°C)	pH 3	pH 5	pH 7	pH 9
K220N	46 ± 0.5	-	-	-	-
K220N, E478P	49 ± 0.1	23 ± 3	93 ± 8	88 ± 5	85 ± 1
K220N, E478P, T484P	47 ±0.1	15 ± 1	93 ± 2	100 ± 1	84 ± 2
K220N, S446P, E478P	43 ± 0.2	32 ± 1	95 ± 2	92 ± 1	83 ± 1

## Data Availability

The data presented in this study are contained within this article and supplementary materials.

## Supplementary Materials

The following are available online at https://www.mdpi.com/1422-0067/22/3/1157/s1, Figure S1: SDS-PAGE of purified ApL variants before (lane 1) and after (lane 2) removal of N255 site, showing the partial deglycosylation of the enzyme (theorical molecular weigth 50 KDa). Two other more putative *N*-glycosylation sites in ApL would explain the remaining glycosylation observed in lane 2. Same amounts of both purified enzymes were utilised. Table S1: Laccase activities with ABTS pH 3 and OD600 of the liquid cultures of *S. cerevisiae* expressing non-deglycosylated and deglycosylated (NGly255) variants of ApL at 20 °C. Table S2: Kinetic constants of purified non-deglycosylated and deglycosylated (NGly255) variants of ApL for the oxidation of ABTS, pH 3. Table S3. Sequences of the primers used. Mutated codons appear underlined.
