# Supplementary material for: Protein Engineering Approaches to Enhance Fungal Laccase Production in S. cerevisiae
Source: Int J Mol Sci. 2021 Jan 25;22(3):1157. doi: 10.3390/ijms22031157 (PMC7866195; doi:10.3390/ijms22031157)
Supplement: Supplementary file 1 [file ijms-22-01157-s001.pdf]

Table S1. Laccase activities towards ABTS pH 3 and OD600 of the liquid cultures of *S. cerevisiae* expressing non-deglycosylated and deglycosylated (NGly255) variants of ApL at 20°C.

Laccase	ABTS U/L	T (°C)	Time (h)	OD600
Non-deglycosylated	778 ± 3	20	120	29 ± 0.1
Deglycosylated (NGly255)	133 ± 3	20	120	30 ± 1

Table S2. Kinetic constants of purified non-deglycosylated and deglycosylated (NGly255) variants of ApL for the oxidation of ABTS, pH3

	K_M (mM)	k_{cat} (s^{-1})	k_{cat} /K_M (s^{-1} mM^{-1})
Non-deglycosylated	0.0045 ± 0.0002	33.26 ± 0.5	7391 ± 346
NGly255	0.0052 ± 0.0002	15.21 ± 0.21	2926 ± 120

Non-deglycosylated and NGly255 ApL variants were grown in 1.2 L EB medium using 1 L flasks as described in 4.5 subsection of M&M. After 120 h of incubation, liquid extracts were filtrated (with filter paper and then using a 0.22 cut off membrane) and concentrated and ultra- diafiltrated using Pellicon cassettes (Merck Millipore, Germany) and Amicon stirred cells (Merck Milli-pore, Germany), both with a 10 kDa cutoff. Laccases were purified by FPLC in two anion exchange steps and one exclusion size step: i) HiPrep QFF 16/10 column in a 100 ml gradient of 0–40% elution buffer; ii) Mono Q HR 5/5 column in a 30 ml gradient of 0–25% elution buffer and iii) exclusion size step with the Superdex 75 column. All columns were purchased from GE Healthcare and the entire purification was performed at pH 7 (Tris-HCl buffer). Enzyme purification was confirmed by SDS-PAGE (12% acrylamide) stained with coomassie blue.

Table S3. Sequences of the primers used. Mutated codons appear underlined.

PeL-pJRoC Rv	5′ ATGCATGCTCGAGCGGCCGCTTACTGGAACTCTGG 3′
ApL-pJRoC Rv	5′ TGCTCGAGCGGCCGCTTATGCAGGAGGAAAAG 3′
ExtFw	5′ CTGGGGTAATTAATCAGCGAAGCGATG 3′
ExtRv	5′ GAGCGTCCCAAAACCTTCTCAAGCAAG 3′
NatFinal-Fw	5′AGGGAAGCCGAAGCAGAATTC3′
NatFinal-Rv	5′GAATTCTGCTTCGGCTTCCCT3′
87Final-FW	5′ CTCTCGAGAAAAGAGAGACTGAAGCTGAATTC 3′
87Final-RV	5′ GAATTCAGCTTCAGTCTCTCTTTTCTCGAGAG 3′
86Final-Fw	5′ GAAGAAGGGGTATCTCTCGAGAAAAGAGGG 3′
86Final-Rv	5′ CCCTCTTTTCTCGAGAGATACCCCTTCTTC 3′
A9D Fw	5′ GGATCATAGGATCCATGAGATTTCCTTCAATTTTTACT**GAT**GTT 3′
A9D Rv	5′ AAC**ATC**AGTAAAAATTGAAGGAAATCTCATGGATCCTATGATCC 3′
PeL K220N Fw	5′ TTCACATTTTCGATTGAT**AAT**CATACC 3′
PeL K220N Rv	5′ AACTCTATGGCGTCAAAGGTATG**ATT**A 3′
PeL T258P Fw	5′ ATAATCGTTAATGCTAATCAG**CCG**ATT 3′
PeL T258P Rv	5′ TATCCAGTAGTTATCAAT**CGG**CTG 3′
PeL S446P Fw	5′ AAT**CCT**GGCGCTTGGTTCTTACAT 3′
PeL S446P Rv	5′ ATGTAAGAACCAAGCGCC**AGG**ATT 3′
PeL E478P Fw	5′ GCTGAAGTTAATGAAGGT**CCG**CAA 3′
PeL E478P Rv	5′ AGTGACTATCTGAGCTTG**CGG**ACC 3′
PeL T484P Fw	5′ GAGCAAGCTCAGATAGTC**CCT**CAA 3′
PeL T484P Rv	5′ CAATGCACGCCAATCTTG**AGG**GAC 3′
ApL NGly255 Fw	5′ GTTGACTACAAAC**CAACCAGTT**GGAGATGGTAATTTCTGG 3′
ApL NGly255 Rv	5′ CCAGAAATTACCATCTCC**AACTGGTTG**GTTTGTAGTCAAC 3′

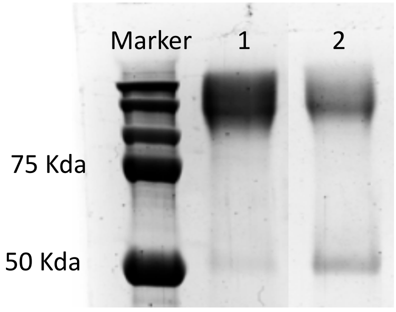

Figure S1. SDS-PAGE of purified ApL variants before (lane 1) and after (lane 2) removal of N255 site, showing the partial deglycosylation of the enzyme (theorical molecular weigth 50 KDa). Two other more putative N-glycosylation sites in ApL would explain the remaining glycosylation observed in lane 2. Same amounts of both purified enzymes were utilised.
